# Global identification of a marine diatom long noncoding natural antisense transcripts (NATs) and their response to phosphate fluctuations

**DOI:** 10.1038/s41598-020-71002-0

**Published:** 2020-08-24

**Authors:** Maria Helena Cruz de Carvalho, Chris Bowler

**Affiliations:** 1 Institut de Biologie de L’Ecole normale supérieure (IBENS), Ecole normale supérieure, CNRS, INSERM, Université PSL, 75005 Paris, France; 2 Faculté des sciences et technologie, Université Paris Est-Créteil (UPEC), 94000 Créteil, France

**Keywords:** Plant cell biology, Non-coding RNAs, Transcriptomics

## Abstract

Often ignored and regarded as mere transcriptional noise, long noncoding RNAs (lncRNAs) are starting to be considered key regulators of gene expression across the Eukarya domain of life. In the model diatom *Phaeodactylum tricornutum*, we have previously reported the occurrence of 1,510 intergenic lncRNAs (lincRNAs), many of which displaying specific patterns of expression under phosphate fluctuation (Pi). Using strand-specific RNA-sequencing data we now expand the repertoire of *P. tricornutum* lncRNAs by identifying 2,628 novel natural antisense transcripts (NATs) that cover 21.5% of the annotated genomic loci. We found that NAT expression is tightly regulated by phosphate depletion and other naturally occurring environmental stresses. Furthermore, we identified 121 phosphate stress responsive NAT-mRNA pairs, the great majority of which showing a positive correlation (concordant pairs) and a small fraction with negative correlation (discordant pairs). Taken together our results show that NATs are highly abundant transcripts in *P. tricornutum* and that their expression is under tight regulation by nutrient and environmental stresses. Furthermore, our results suggest that in *P. tricornutum* Pi stress response NAT pairs predominantly regulate positively the expression of their cognate sense genes, the latter being involved in several biological processes underlying the control of cellular homeostasis under stress.

## Introduction

Diatoms are unicellular microalgae belonging to the stramenopile supergroup that emerged from an ancient secondary endosymbiosis over 200 My ago^1,2^. They have since risen to prominence in the global ocean and are now amongst the major ocean primary producers^3–5^. Very good competitors when nutrients are abundant^6^, diatoms are also extremely resilient to nutrient fluctuations in their environment, being able to quickly resume growth as soon as the environment is again favorable^7–9^. Understanding of the molecular underpinnings regulating this resilience is nonetheless still very incomplete.

Long noncoding RNA (lncRNA) transcripts originate from unannotated regions of genomes and were longtime regarded as transcriptional noise. However, with the huge amount of data generated by next generation high throughput RNA sequencing, and subsequent functional characterization of several lncRNAs, they are now gaining momentum as key regulators of gene expression across the Eukarya domain of life. LncRNAs form a heterogeneous class of transcripts that are generally capped and polyadenylated, like mRNA transcripts, but have as common features a sequence longer than 200 nucleotides with no viable open reading frame (ORF)^10,11^. Based on their relationship with protein coding genes, lncRNAs can be called intergenic (lincRNAs) when they are located between protein coding genes, intronic (incRNAs) when they are expressed from introns, or natural antisense transcripts (NATs, also referred to as *cis*-NATs) when they are expressed from the opposite strand of sense genes of the same genomic locus^10^. The latter group seems to be by far the most pervasive, having been described in both prokaryotes^12^ and eukaryotes^13^. Historically, NATs have been neglected as byproducts of spurious transcription but enough evidence has now been gathered to show that NATs can de facto be important modulators of gene expression. NATs function by several different mechanisms, either locally, around the locus where they are expressed, or far from the site of transcription^14^. NATs may affect directly their cognate sense gene expression by transcription interference^15^, or indirectly by assisting chromatin remodeling^16–19^. NATs may also form double stranded regulatory RNAs with their overlapping cognate transcripts and be processed by Dicer or Dicer-like enzymes, leading to RNA interference and gene silencing^20–22^, or be involved in RNA editing and/or RNA masking^14^. Downstream effects of RNA masking include alternative splicing^23^ and regulation of translation^24,25^. Tissue- or developmental-specific expression of NATs seems to be an important determinant of the regulatory role of some NATs^13^. These noncoding transcripts have also been reported to be involved in the regulation of responses to various environmental signals, from yeast, to plants, to humans^26–30^.

In the model pennate diatom, *Phaeodactylum tricornutum*, we have previously reported the occurrence of ~ 1,500 lincRNAs, many of which being under phosphate (Pi), nitrogen (N)^9^ or *p*CO_2_^31^ regulation. Using strand-specific RNA-sequencing data^9^ we now expand the repertoire of *P. tricornutum* lncRNAs by identifying 2,628 novel NATs and characterizing their expression patterns under phosphate fluctuations as well as under other environmental constraints. Amongst the -Pi stress regulated NATs, approximately one third were correlated with their cognate sense protein coding genes, forming tight sense-antisense NAT-mRNA pairs (NAT pairs). Interestingly, the majority of the identified NAT pairs were positively correlated, forming concordant pairs, with only a small fraction being negatively correlated, forming discordant pairs. These results suggest a new level of gene regulation in *P. tricornutum* whereby NAT pairs predominantly regulate positively the expression of their cognate sense protein coding genes.

## Materials and methods

### Diatom culture conditions

Axenic cultures of *P. tricornutum* strain CCMP632 were obtained from the Center for the Culture of Marine Phytoplankton (Maine, USA). They were cultured in 250 mL filtered (Grade 1 Whatman) steam-sterilized artificial sea water (40 g/L, Sigma) supplemented with f/2 nutrients, elements and vitamins^32^ with the exception of silica (f/2-Si), in 1 L glass flasks under continuous shaking (100 rpm) at 20 °C under cool white fluorescent lights at 100 µmol.m^-2^ s^−1^ with a 12 h photoperiod. For phosphate fluctuation studies we used the 5 physiological time points previously described^9^. Equal aliquots of 4 day-old cultures from the same batch culture were inoculated in parallel in 250 mL fresh f/2-Si media (control conditions) and in 250 mL fresh f/2-Si media without phosphate supplement (Pi depleted) and cultured in the same conditions as described above. For Pi re-supplementation, 4-d-old Pi-depleted cultures were pelleted by centrifugation for 10 min at 1,500 g, resuspended in 250 ml of fresh f/2-Si medium and cultured in the same conditions described above. For nitrogen depletion studies, equal aliquots of 4 day-old cultures from the same batch culture were inoculated in parallel in 250 mL fresh f/2-Si media (control conditions) and in 250 mL fresh f/2-Si media without nitrogen supplement (N depleted) and cultured in the same conditions as described above. For heat and cold stress treatments, cultures were transferred for 20 min to a water bath at 30 °C and 10 °C, respectively. Salt shock treatment consisted in cultures being transferred to f/2 media with twice the amount of sea salt concentration (80 g/L) for 20 min. Culture growth was followed using a hematocytometer (Fisher Scientific, USA). Cultures (controlled and treated) were all started with the same initial cell densities of ~ 4.5 × 10^5^ cells/mL. Cells were harvested at the same time of day (6 h after beginning of the light period) by vacuum filtration on 0.2 µm polycarbonate filters (Millipore, United States), flash-frozen in liquid nitrogen and maintained at − 80 °C until used. All the experiments were repeated at least 3 times.

### Data sources

The strand-specific RNA Sequencing (ssRNA-seq) datasets used in this study were obtained from a previous publication^9^. Briefly, strand-specific (ss)RNA-seq reads were mapped to the *P. tricornutum* genome using TopHat (version 2.0.8)^33^ and then assembled using Cufflinks (v2.1.1)^34^. Each ssRNA-seq sample was then merged and annotated using Cuffcompare (v2.1.1)^34^. Expression levels of each gene were then calculated by the fragments per kilobase of exons per million fragments mapped (FPKM) using Cuffdiff (v2.1.1)^34^. The Pearson correlation coefficients (PCC) were calculated between the FPKM of 2–3 replicates of each 5 physiological states corresponding to 2–3 independent experiments. In order to increase the depth of the reads for increased detection of noncoding RNA transcripts, and since the PCCs were high (> 0.9), we pooled reads derived from the two most robust replicates as previously described^9^. A twofold variance in FPKM and a *P* value < 0.05 (Fisher’s exact test) were used as cutoff to define differentially expressed (DE) genes.

### Quantitative real-time reverse transcription PCR

Total RNA was extracted from *P. tricornutum* flash frozen cell pellets using the Trizol method according to the manufacturer’s instructions (Invitrogen). Extracted RNA was treated with TurboDNAse I (Life Technologies AM2238) and then purified using and RNeasy spin column (Qiagen) with 0.5 volumes of ethanol, washed and then eluted with 50 μL room temperature molecular grade water (Qiagen). The quality of the purified RNA was assessed using the Agilent 2100 Bionanalyzer. For cDNA synthesis, 500 ng RNA was incubated with SuperScript III Reverse Transcriptase (Invitrogen) according to the manufacturer’s instructions. For quantitative RT-PCR analysis, cDNA was amplified using SYBR Premix ExTaq (Takara) with specific primers (Table S2) picked from a random sample of identified NATs. Primers were designed with the Primer-Blast program (https://www.ncbi.nlm.nih.gov/tools/primer-blast/) defining PCR amplicon size between 70–150 bp and *Phaeodactylum tricornutum* CCAP 1055/1 (taxid: 556484) as the reference organism to check for primer pair specificity. Quantitative 2-step PCR conditions were set as following: 95 °C for 10 s followed by 40 cycles of 95 °C for 5 s and 60 °C for 30 s and a final cycle of 95 °C for 10 s and 60 °C for 5 s in a Bio-Rad CFX96 real-time system or a Roche LightCycler 480 in a 384 well. *CDKA* and *HISTONE 4* mRNA levels were used for normalization^35^.

### Identification of NATs and phosphate-regulated NAT-mRNA pairs

All assembled intergenic transcription units were collected as lncRNA candidates. Candidates with length ≥ 200 nt, predicted ORF ≤ 100 amino acids and with at least 50 nt overlap with annotated gene models were defined as NATs^30,36^. Low abundance NATs (FPKMmax < 1) were excluded from the analysis. The length of transcripts was used to compare transcript length distribution. For ORF predictions, the spliced transcripts were used and sent to GenScan^37^.

To detect putative NAT-mRNA pairs (expression correlation between sense-antisense NAT and overlapping gene models), the PCCs were calculated using FPKMs of all the phosphate-responsive NATs versus mRNAs encoded by their overlapping genes. We classified phosphate-regulated NAT pairs into two groups: concordant regulation and discordant regulation. For the identification of phosphate-regulated concordant NAT pairs, one transcript should change in expression level in the same direction as its partner during phosphate fluctuations and for discordant NAT pairs, expression levels of the sense and antisense transcripts should change in the opposite direction. Only those Pi responsive NAT-mRNA pairs with r^2^ ≥ 0.6 (positive or negative correlation) were considered as putative NAT pairs.

## Results

### Genome wide identification of NATs in *Phaeodactylum tricornutum*

**Figure 1:**
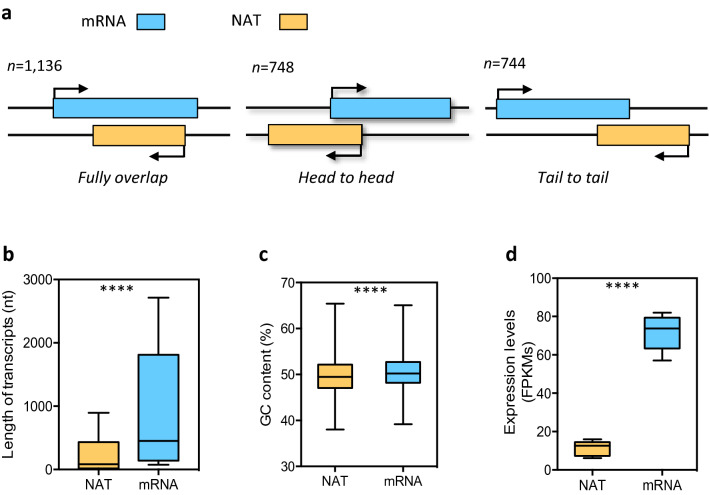
Characteristics of NAT and mRNA transcripts in *Phaeodactylum tricornutum*. (**a**) Schematic representation of the long noncoding natural antisense transcripts (NAT) identified in *P. tricornutum*. NATs include transcripts that are fully overlapped by their cognate protein coding genes (mRNA), or that have partial overlap at the 5′ (head to head) or the 3′ (tail to tail) ends. Arrows indicate the direction of transcription; n, number of NATs identified in each category. (**b**) length distribution in nucleotides (nt) (**c**) GC content (%) and (**d**) expression levels detected by RNA-Seq (FPKMs) of NATs and mRNAs. Statistical difference between NATs and mRNAs was assessed by a paired *t* test, (****) *P value* < 0.0001.

Using high-depth strand-specific RNA sequencing (ssRNA-seq) data from at least 10 independent cDNA libraries from polyadenylated [poly(A) +] RNAs under normal or phosphate stress conditions (Accession no. GSE669970)^9^, we assembled all the reads to reconstruct the *P. tricornutum* transcriptome. After removing transcripts that were previously annotated as protein coding, transcripts with short length (< 200 nt), low abundance (FPKMmax < 1) and putative open reading frames (ORFs) of more than 100 amino acids, we identified a total of 4,138 lncRNAs. Amongst these were the 1,510 previously annotated lincRNAs^9^ plus 2,628 newly identified transcripts belonging to the natural antisense transcript family (NATs) (Supplementary Table S1), having at least 50 nt overlapped with annotated gene models (Phatr3). Around 43% (1,136) of the NATs identified were transcripts that fully overlapped the coding genes on the opposite strand and the remaining 57% (1,492), were partial overlaps, either head to head (748) or tail to tail (744) (Fig. 1a). Overall, the newly identified NATs cover 21.5% of the annotated genomic loci (Phatr3).

### NATs in *P. tricornutum* represent cross-kingdom genomic features

We analyzed the average length and expression levels of the identified NATs and compared these to the annotated mRNAs detected in our transcriptomic data. NATs were significantly shorter in length than mRNAs (Fig. 1b) (*P* value < 0.0001, paired *t* test) with a median length of 740 nt for NATs and 1,266 nt for mRNAs. Similarly, lincRNAs were also found to be shorter than mRNAs^9^. Interestingly, previously identified *P. tricornutum* lincRNAs which have a median size of 482 nt were significantly shorter than NATs (Supplementary Fig. S1). Another feature of functional lncRNAs is lower GC content than mRNAs^38^, which was noted previously in *P. tricornutum* lincRNAs^9^ and was also found in *P. tricornutum* NATs (Fig. 1c). The expression levels of NATs and mRNAs, estimated by FPKM using Cuffdiff^34^, revealed that the average expression levels of NATs was much lower than those of protein coding mRNAs (*P*-value < 0.0001, paired *t* test) (Fig. 1d). Interestingly, significant differences in expression levels were found between NATs and lincRNAs in *P. tricornutum*, with NATs having the lowest expression levels of the three transcript categories (NATs, lincRNAs, mRNAs) (Supplementary Fig. S1).

### NATs are highly abundant in the *P. tricornutum* phosphate responsive transcriptome

**Figure 2:**
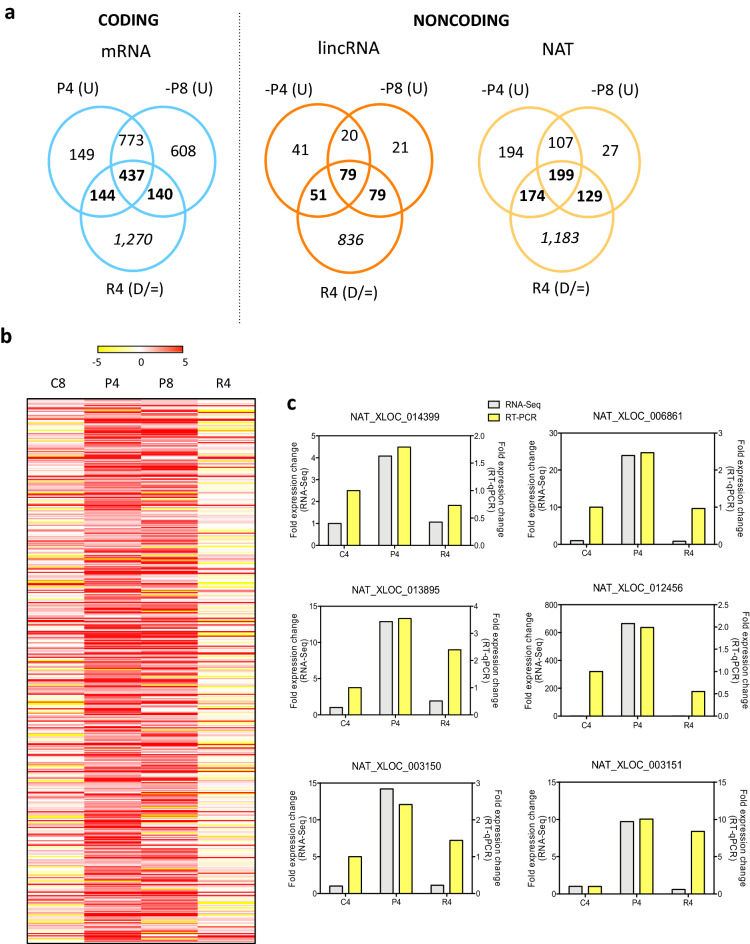
*P. tricornutum* NAT detection and expression under Pi fluctuations. (**a**) Venns representing the three categories of phosphate responsive *P. tricornutum transcripts*: mRNA (coding), NAT and lincRNA (noncoding) under phosphate fluctuations. (**b**) Heatmap of the NATs up-regulated by Pi depletion (> twofold, *P* value < 0.05). Rows represent log2 expression change in relation to control 4 days, columns represent four different physiological states marked by Pi fluctuations (**c**) RT-qPCR validation of NAT RNA-Seq expression data under Pi fluctuation. P4, 4 days Pi stress; P8, 8 days phosphate stress; U, up-regulated; R4, 4 days recovery after 4 days Pi stress; D, down-regulated or = , unchanged transcripts.

Diatoms are frequently subjected to nutrient fluctuations in their environment, and phosphate has been shown to be amongst the major nutrients affecting primary production in the ocean, namely in coastal areas where *P. tricornutum* is predominantly present^39,40^. In order to assess the functional relevance of the identified NATs in *P. tricornutum* we sought to investigate their pattern of expression under Pi fluctuations. In order to do this we calculated the FPKM from the ssRNA-seq datasets obtained from diatom cultures under different Pi regimes (Pi starved for 4 and 8 days, Pi resupplemented for 4 days after 4 days depletion and control cultures grown for 4 and 8 days in full media) ^9^. Of the 2,628 NATs identified, 1,481 NATs were differentially expressed (DE) (fold change ≥ 2 or ≤ 0.5, *P* value < 0.05) under Pi depletion, which corresponds to ~ 56% of all the NATs identified in *P. tricornutum.* Amongst these DE NATs, 830 were up-regulated (fold change ≥ 2, *P* *value* < 0.05) in response to Pi depletion (after 4 and/or 8 days Pi depleted) and 402 were specifically up-regulated by Pi since once Pi was re-supplied to the media, their expression levels returned to control values or lower (Fig. 2a,b; Supplementary Table S1). These results were validated by repeating Pi fluctuation experiments (Pi depletion and re-supply), extracting RNA from the cultures and performing RT-qPCR using specific primers of a randomly selected set of Pi depletion up-regulated NATs (Supplementary Table S2) and obtaining equivalent expression trend variation (Fig. 2c). Regarding Pi-specific regulation in the other gene categories, we detected 720 mRNAs and 209 lincRNAs (Fig. 2a). This reveals that the Pi-regulated noncoding transcriptome (NATs and lincRNAs) in *P. tricornutum* is almost equivalent in number to the Pi-regulated coding transcriptome (mRNAs) with 611 and 720 transcripts, respectively, with NATs being the dominant lncRNAs transcripts.

### NAT expression is regulated by environmental stress

**Figure 3:**
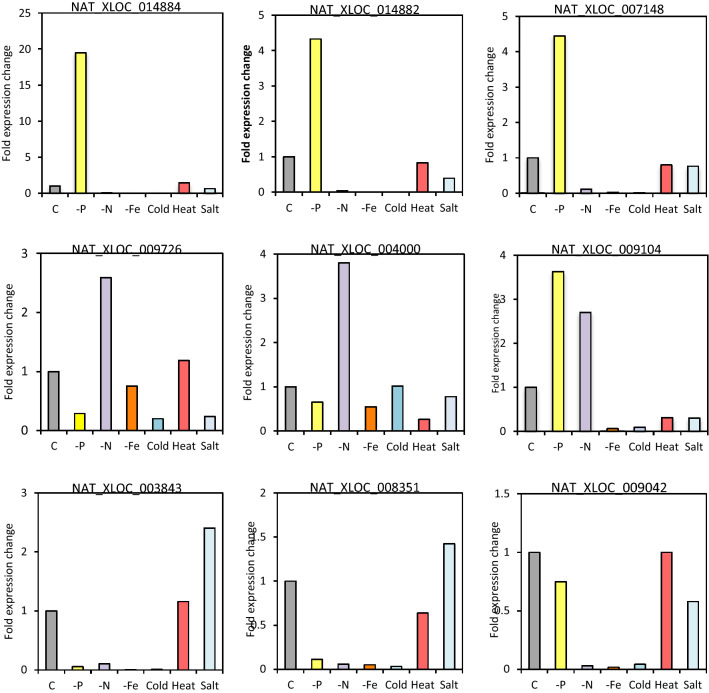
RT-qPCR stress expression analysis of NATs in *P. tricornutum* cultures submitted to different treatments mimicking recurrent environmental variations. Grown in C, full media; -P, media without phosphate; -N, media without nitrate; -Fe, media without iron; Cold, submitted to 10 °C for 20 min; Heat, submitted to 30 °C for 20 min; Salt, submitted to 2 × salt in media for 1 h. All cultures were 4 days old at the time of sampling.

To further validate our results and investigate whether NATs in *P. tricornutum* were responsive to other stresses besides phosphate stress, we checked by RT-qPCR the relative expression levels of a randomly selected set of NATs using cDNA libraries originating from *P. tricornutum* cultures submitted to different treatments. The treatments were made with the intent to mimic recurrent nutrient or mineral stresses, such as nitrogen and iron depletion over the course of 4 days, thermal and salt stress designed to mimic changes in environmental temperature or salinity due to vertical mixing of currents. All the NATs tested were found to be stress-regulated either with an increased expression or a reduced expression, depending on the stress (Fig. 3). NAT-XLOC_014884, NAT_XLOC_014882 and NAT_XLOC_007148 were all up-regulated by Pi depletion but down-regulated under N or Fe depletion (Fig. 3). On the other hand, NAT_XLOC_009104 was up-regulated by Pi and also N depletion, suggesting it could be involved in common pathways shared by the two nutrient stresses, whereas NAT_XLOC_009726 and NAT_XLOC_004000 were up-regulated by N depletion only. This confirms the specificity of the NAT response and shows that despite being expressed at lower levels than mRNAs (Fig. 1d), diatom NATs are under tight environmental regulation and are not randomly expressed. Given the specificity and robustness of the response, it is unlikely that the noncoding transcriptome and, in particular, the novel NATs detected in the present study, are transcriptional noise or byproducts of spurious transcription.

### NAT-mRNA pairs in *Phaeodactylum* are predominantly concordant

**Figure 4:**
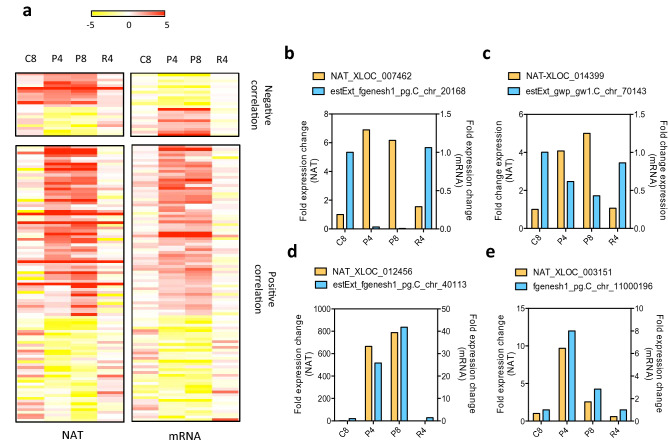
NAT-mRNAs pairs identified in *P. tricornutum* under Pi fluctuation. (**a**) Heat map of log2 fold expression changes under Pi fluctuation of the NAT-mRNA pairs (with negative or positive correlation) identified in *P. tricornutum* (r^2^ > 0.6, fold change ≥ 2 or ≤ 0.5, *P *value < 0.05). (**b,c**) fold expression change of two discordant NAT-mRNA pairs; (**d**,**e**) fold expression change of two concordant lncNAT-mRNA pairs. P4, 4 days Pi depleted; P8, 8 days Pi depleted; R4, 4 days Pi resupply after 4 days Pi depletion; C8, 8 days control conditions.

To detect putative expression correlations between sense-antisense NATs and overlapping gene models forming NAT (-mRNA) pairs, we calculated the Pearson correlation coefficients (PCCs) using the FPKMs of all the responsive NATs versus the mRNAs encoded by their overlapping genes. We defined Pi stress regulated NATs if their expression responded to the following pattern: up-regulation (fold change ≥ 2, *P* value < 0.05) in Pi depleted conditions with subsequent down-regulation (fold change ≤ 0.5, *P* value < 0.05) or return to control levels when Pi was resupplied to the media or the opposite; down-regulation (fold change ≥ 0.5, *P* value < 0.05) in Pi depleted conditions with subsequent up-regulation (fold change ≤ 2, *P* value < 0.05) or return to control levels when Pi was resupplied to the media. We defined concordant NAT pairs as those in which the expression levels of both transcripts changed in the same direction during phosphate fluctuations and for discordant NAT pairs, the expression levels of the two transcripts should change in opposite directions. We found that amongst the 810 Pi stress regulated NATs, 121 formed sense-antisense NAT pairs which reveals interdependency between both transcripts (Fig. 4). Correlated NAT-mRNA pairs included 34 tail-to-tail, 39 head-to-head and 48 fully overlapped (Supplementary Table S3). Amongst these interdependent NAT pairs, the majority were positively correlated forming concordant NAT pairs (99) and only a small fraction were discordant (22) (Fig. 4a, Supplementary Table S3). Within the concordant pairs, 25 were tail-to-tail, 36 head-to-head, and 38 were fully overlapping NAT-mRNA pairs. Within the discordant pairs, 9 were tail-to-tail, 3 head-to-head and 10 fully overlapped, showing that 5′end pairing is less frequent for discordant NAT-pairs in *P. tricornutum* than tail-to-tail and fully overlapping pairs.

### Insights into the putative regulatory functions of the Pi responsive NAT-mRNA pairs

We performed a search on the available gene ontology (GO) terms of the annotated transcripts with a correlated NAT (NAT-mRNA pairs with positive and negative correlation). Amongst the most abundant GO annotation categories were genes involved in the regulation of transcription or DNA-binding (Supplementary Table S4). These included a gene coding for a heat shock factor (HSF, 34415) which was upregulated under Pi stress and was negatively correlated to its associated NAT, a gene coding for a basic-leucine zipper (bZIP) transcription factor (47278) and a PHD-finger protein (44901), both of which being upregulated under Pi stress, accompanied by their respective cognate NATs (Supplementary Table S4). A mRNA coding for Histone 3 protein (50695) also formed a concordant NAT pair, which was upregulated under Pi stress. Transporter-related mRNAs were also present forming five concordant NAT pairs, four of which were upregulated under Pi stress (Supplementary Table S4). Amongst these was a mRNA coding for a phosphate transporter (40433) which has been previously shown to be Pi deficiency-specific^9^. The present data adds another layer of information, suggesting that this transporter gene could be under the positive control of its concordant NAT and supports an intricate role for the NAT-mRNA pair in phosphate homeostasis regulation under Pi stress. Another GO annotation category well represented amongst the NAT pairs were genes coding for metabolism-related proteins, mainly protein metabolism (Supplementary Table S4). Interestingly, these formed mainly discordant NAT pairs with the mRNAs being downregulated while their associated NATs were upregulated in response to Pi depletion (Supplementary Table S4. Genes involved in signaling pathways were also detected, including two mRNAs coding for a putative protein containing a SET domain (36270) and one for a putative protein kinase (43421) (Supplementary Table S4).

## Discussion

Diatoms are the most abundant eukaryotic phytoplankton in the contemporary ocean. One of the main reasons behind their ecological success is their extreme resilience to a changing environment, enabling them to quickly shift between states of exponential division under favorable conditions, to quiescent states when the environment is less favorable, and then back to exponential cell division when the environment is again favorable. Underpinning this ability is a highly responsive and dynamic molecular system regulating cellular function and metabolism in an extremely efficient way.

Long noncoding RNAs (lncRNAs) are transcripts that have been assigned many regulatory functions across the Eukarya domain of life and diatoms are excellent model systems to explore the occurrence and functional relevance of the long noncoding transcriptome in a context of stress resilience. In the present work we show that long noncoding natural antisense transcripts (NATs) are more pervasive in the *P. tricornutum* transcriptome than what was found in previous gene annotation efforts, with 21.5% of the annotated protein coding genes being overlapped by a NAT as opposed to the previously 1% reported^41^. This finding is in agreement with previous reports in several other eukaryotic genomes revealing the prevalence of NATs, with 27% of genomic loci in yeast^42^, close to 70% in Arabidopsis^30^ and ~ 72% in mouse and human^43^. It is likely that with the increased routine use of strand-specific deep RNA-sequencing techniques, more and more NATs will be uncovered and characterized across all eukaryotes.

NAT expression in *P. tricornutum* is specifically modulated by stress and several NATs were shown to be nutrient or environmental stress-specific (Fig. 4). This shows that the expression of NATs does not occur randomly and is instead highly regulated by external signals, strongly suggesting function. This is further supported by what has been found in other model systems such as yeasts, plants or animals^16,17,24,44–47^. Like the previously characterized lincRNAs^9^, NATs in *P. tricornutum* have significantly lower expression levels than mRNAs (Fig. 1). This is a common cross-kingdom genomic feature of lncRNAs in general, and of NATs in particular, since these transcripts have been consistently shown to be expressed at much lower levels than mRNAs in plants^30,46,48^, humans^49,50^ and yeast^51^. Unlike mRNAs that need to be converted to proteins to be active, NATs can act locally, and eventually at the site of transcription, so it is reasonable to assume that fewer copies of NATs will be necessary to execute a function than those of mRNA. NATs can interact with DNA either directly through RNA–DNA interaction or indirectly through RNA-chromatin interactions^42^. For instance the NAT of CDKN1A, a tumor suppressor gene in humans, has been shown to recruit an enzymatic regulatory complex that induces H3K27me3 to suppress the sense promoter region^47^. Another example is MAS, an antisense NAT of MADS AFFECTING FLOWERING 4 (MAF4), that mediates the recruitment of WDR5a leading to enhanced H3K4me3 in the MAF4 locus in *Arabidopsis*^46^. Such local actions would not require a high number of transcripts to achieve function. In *P. tricornutum* we detected several NAT pairs that could be involved in the regulation of transcription, metabolism, signaling pathways and phosphate homeostasis in response to Pi depletion (Supplementary Table S4).

Despite being expressed at lower levels than mRNAs, NATs have been shown to be expressed in a very specific manner^13^. This is a common feature of lncRNAs and, interestingly, lncRNAs have also been shown to be comparatively more tissue-specific than mRNAs^52^. In unicellular organisms such as yeast, *Chlamydomonas* and in the present work, diatoms, NAT expression is under tight regulation by environmental signals^44,53^. In *Chlamydomonas* NAT expression has been shown to be regulated in response to sulfur deprivation^53^ and in yeast, NATs have been shown to be regulated by osmotic stress^44^. NAT regulation by environmental stimuli has also been detected in land plants, where NATs have been reported to be induced by different stresses such as salt stress^54^, light^30^, drought and ABA^46^ and cold^55^.

Given the potential to form double-stranded RNA molecules, some diatom NATs may be the precursors of short interfering (si)RNAs, leading to gene silencing, as has been demonstrated in plants^21,56^. Although no canonical miRNAs have been detected in diatoms^57,58^, gene silencing through inverted or antisense transcripts is known to occur and is commonly used in functional studies to knock down transcription of target genes^59^. This possibly operates through siRNA production which is supported by the fact that the *P. tricornutum* genome encodes one Dicer-like and an Argonaute protein^59^. It has been largely assumed that sense-antisense overlapping transcripts are processed by Dicer or Dicer-like proteins to produce small interfering RNAs (siRNAs)^20^. However, NATs have been shown to operate in many different ways besides the RNAi pathway. Overall, only a few reports have shown the involvement of NATs in the siRNA gene silencing pathway, suggesting that this is not the major mode of action of NATs^30^.

Transcription interference which, like RNA interference also results in the negative correlation between NAT-pair expression, whereby the expression of one gene down-regulates the expression of the cognate gene on the opposite strand^15^, does not seem to be prevalent in *P. tricornutum* because only a small number of robust discordant NAT pairs were identified (Fig. 4a). This, nonetheless, cannot be excluded to occur in the case of the discordant NAT pairs detected in this study (Fig. 4; Supplemental Table S3). There are several modes of transcriptional interference, all of which suggest a negative regulation of NAT pairs. These include overlapped competing promoters for RNA Pol II occupation, dislodgement of one RNA Pol II by a second RNA Pol II on the opposite strand, occlusion of the initiation start site of one of the strands by RNA Pol II occupation on the other strand and, finally, collision of two RNA Pol II-transcribing opposite strands^14, 15^. The extent of this phenomenon of gene regulation by NATs is still debatable. Interestingly, in *Phaeodactylum* > 80% of the NAT pairs responsive to Pi stress are concordant, which is strikingly different from what has been demonstrated in Arabidopsis where concordant and discordant NAT pairs seem to be equally represented in response to light^30^ or in yeast, where the majority of the NAT pairs identified are discordant^60,61^. This reveals that *Phaeodactylum* Pi responsive NATs predominantly positively regulate the expression of their cognate sense protein coding genes.

In *Arabidopsis* both concordant and discordant NATs pairs have been functionally characterized. For example the NAT FLORE was found to form a discordant NAT pair with CDF5, a member of a plant specific transcription factor with a key regulatory role in connecting the circadian clock to periodic flowering-time control^45^. Another example of a well characterized discordant NAT pair function is the epigenetic silencing of FLOWERING LOCUS C (FLC) by its NAT, COOLAIR^18^. On the other hand, MAS forms a concordant NAT pair with MAF4 and during vernalization leads to enhanced H3K4me3 in the MAF4 locus resulting in increased expression^46^. A recent work using single-molecule RNA FISH revealed that the discordant sense-antisense transcripts, COOLAIR and FLC could co-occur in the same cell, but were mutually exclusive at individual loci^61^. It will thus be interesting to investigate the transcription patterns of the Pi responsive NAT pairs in *P. tricornutum* at the single-cell level.

The cells of multicellular eukaryotes benefit from the protective advantages of complex homeostasis. By contrast, unicellular diatoms are subject to constant environmental change which affects their entire organism. The speed at which a unicellular species can respond to its environment directly determines its chances for survival^62^. It is therefore likely that unicellular species employ an intricate regulatory network allowing for a rapid shift between processes leading to adaptation in response to environmental signals. Amongst the NAT pairs responsive to Pi fluctuations in *P. tricornutum* were mRNAs coding for proteins involved in signaling pathways, transcription regulation and phosphate transport. This suggests an important role for NATs in the orchestration of the Pi stress response.

In summary, with this work we show that NATs, with their highly specific expression profiles, could be major actors of cellular processes, particularly serving to regulate gene networks in stress adaptation pathways in *P. tricornutum*. Functional studies currently underway are likely to provide further information and novel insights on the importance of NATs in this marine diatom environmental stress response and resilience to a changing environment.

## Supplementary information

Supplementary information 1.

Supplementary information 2.

## Data Availability

The sequencing data used in this study is available at the Gene Expression Omnibus (GEO) database https://www.ncbi.nlm.nih.gov/geo (Accession No. GSE66997).
