# Supplementary material for: Global identification of a marine diatom long noncoding natural antisense transcripts (NATs) and their response to phosphate fluctuations
Source: Sci Rep. 2020 Aug 24;10:14110. doi: 10.1038/s41598-020-71002-0 (PMC7445176; doi:10.1038/s41598-020-71002-0)
Supplement: Supplementary file 1 — Supplementary information 1. [file 41598_2020_71002_MOESM1_ESM.pdf]

Global identification of a marine diatom long noncoding natural antisense transcripts (NATs) and their response to phosphate fluctuations

Maria Helena Cruz de Carvalho[1,2*], Chris Bowler[1]

[1]Institut de Biologie de l'ENS (IBENS), Département de biologie, École normale supérieure, CNRS, INSERM, Université PSL, 75005 Paris, France.

[2]Université Paris Est-Créteil (UPEC), Faculté des Sciences et Technologie, 61, avenue du Général De Gaulle 94000 Créteil, France

Correspondence: Maria Helena Cruz de Carvalho
cruz@biologie.ens.fr

SUPPLEMENTARY DATA

Figure S1 |Features of lincRNA, NAT and mRNA transcripts in P. tricornutum. (a) length frequency distribution in nucleotides (nt) (b) mean length in nucleotides (nt) and (c) expression levels detected by RNA-Seq (FPKMs) of lncNATs and mRNAs. Different letters translate significant statistical difference as assessed by Tukey's multiple comparison test (P value < 0.05).

Table S1- Phaeodactylum tricornutum NAT candidates

Table S2- Primers for RT-qPCR

Table S3- Phaeodactylum tricornutum Pi responsive NAT pair candidates

Table S4- GO terms associated to Pi responsive NAT-mRNA pairs

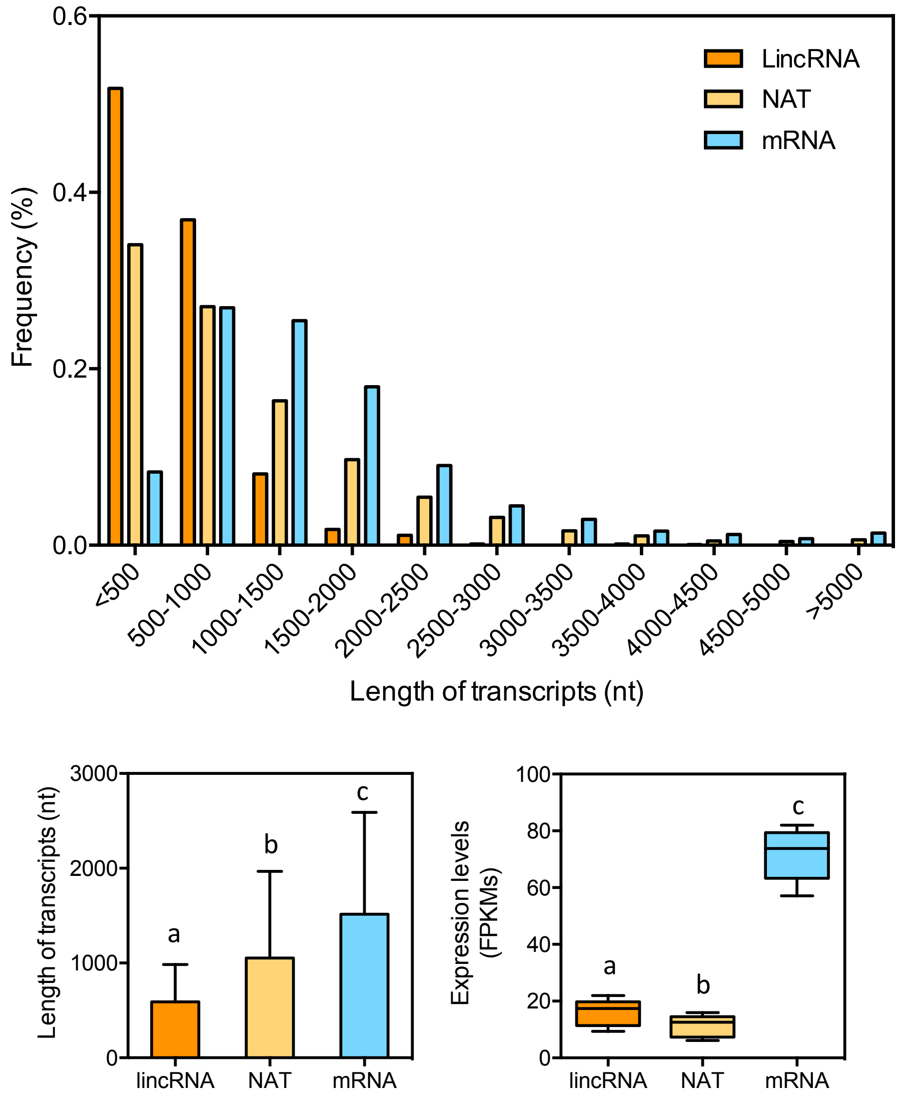

Figure S1 |Features of lincRNA, NAT and mRNA transcripts in *P. tricornutum*. (a) length frequency distribution in nucleotides (nt) (b) mean length in nucleotides (nt) and (c) expression levels detected by RNA-Seq (FPKMs) of lncNATs and mRNAs. Different letters translate significant statistical difference as assessed by Tukey's multiple comparison test (*P* value < 0.05.
